# **γδ** T Cells Cross-Link Innate and Adaptive Immunity in *Mycobacterium tuberculosis* Infection

**DOI:** 10.1155/2011/587315

**Published:** 2011-01-05

**Authors:** Serena Meraviglia, Sary El Daker, Francesco Dieli, Federico Martini, Angelo Martino

**Affiliations:** ^1^Dipartimento di Biopatologia e Biotecnologie Mediche e Forensi, Università di Palermo, 90134 Palermo, Italy; ^2^Laboratory of Cellular Immunology, National Institute for Infectious Diseases “Lazzaro Spallanzani” IRCCS, Via Portuense 292, 00149 Rome, Italy

## Abstract

Protective immunity against mycobacterial infections such as *Mycobacterium tuberculosis* is mediated by interactions between specific T cells and activated antigen presenting cells. To date, many aspects of mycobacterial immunity have shown that innate cells could be the key elements that substantially may influence the subsequent adaptive host response. During the early phases of infection, innate lymphocyte subsets play a pivotal role in this context. Here we summarize the findings of recent investigations on **γδ** T lymphocytes and their role in tuberculosis immunity.

## 1. Introduction

Tuberculosis (TB), caused by *Mycobacterium tuberculosis* (MTB), is one of the most prevalent and commonest serious infectious diseases worldwide, afflicting almost 10 million people annually [1]. The disease, fuelled by Human Immunodeficiency virus (HIV) infection and poverty, is out of control in developing countries, and the emergence of drug-resistant strains threatens TB control in several other regions of the world [1, 2]. The current available vaccine, Bacillus Calmette-Guerin (BCG) as well as existing therapeutic interventions for TB, are at present suboptimal. Thus, new vaccines and immunotherapeutic strategies are urgently required to improve TB control efforts [3]. A better understanding of the immunopathogenesis of TB could facilitate the identification of correlates of immune protection, the design of effective vaccines, the rational selection of immunotherapeutic agents, and the evaluation of new drug or adjuvant candidates [3, 4].

Generally, effective immune responses to pathogenic and commensal microorganisms require T lymphocytes be endowed with effector properties appropriate to each challenge. In this context, CD4 T lymphocytes differentiate in the peripheral tissues to adopt a variety of fates such as the T helper (Th)-1 cells, which produce interferon (IFN)-*γ* and Th-2 cells, which produce interleukin (IL)-4. Specific cell-mediated immunity is critical in the host defense against mycobacteria, but many aspects of mycobacterial immunity involve other levels of responses. TB is primarily a disease of the lung, and dissemination of the disease depends on productive infection of this critical organ. Upon aerosol infection with MTB, the acquired cellular immune response is slow to be induced and to be expressed within the lung. MTB has a variety of surface molecules and soluble products that interact with the innate immune compartment, and this interaction along with the autoregulation of the immune response by several mechanisms results in less-than-optimal control of bacterial growth. Antigen-specific *γδ* T cells represent an early innate defense that may play a role in antimycobacterial immunity. Studies done in humans and animal models have demonstrated complex patterns of *γδ* T cell immune responses during early mycobacterial infections and chronic TB. In this paper, we focus on the role of *γδ* T cells in the innate defense and the immune regulation of mycobacterial immunity, as well as on their possible involvement in the new immunotherapies.

## 2. *γδ* T Cells: An Overview

The concept of a strict dependent relationship between cells from innate and adaptive immunity changed the point of view about the regulation of immune system. During the most part of host reactions, both adaptive and innate sections cooperate in the host's protection and tissue damage. However, the infection by microbial agents often occurs in the peripheral tissues whereas specific naïve T lymphocytes are confined to lymphoid areas. Thus, the innate cells recruited or resident in the tissues play a crucial role in the containment of infection and the deployment of adaptive immune response [5]. Like *αβ* T lymphocytes, *γδ* T cells carry antigen T-cell receptors (TCR) that vary in the physical properties of their ligand-binding sites [6]. Indeed, *γδ* TCR have a great potential of diversity at their putative ligand-binding sites as well as *αβ* T and B cells. This means that *γδ* T cells have a potential to recognize different pathogenic agents through the recognition of common molecular patterns.

*γδ* T cells constitute a whole system of functionally specialized subsets that have been implicated in the innate responses against tumors and pathogens, the regulation of immune responses, cell recruitment and activation, and tissue repair [7]. The concept of *γδ* T cells as “first line of defense” has been recently reviewed as a nonredundant system of responses based on an innate immunity program involved in systemic and specific responses depending on the inflammatory microenvironment, on microbes features and on signals that are engaged. This concept address facets of a complex behaviour where several enigmas started to be resolved. In humans and other primates, *γδ* T cells represent a small percentage among peripheral blood lymphocytes (1–5%) and represent a special case of CD3+ T cells relying on their known separate set of receptor genes [8]. Thus, *γδ* T cells are a specialized and independent population of lymphocytes, and basing on TCR recombination different settings of *γδ* T cells may be now classified. The first *γδ* T cell lineage appearing in the human foetal thymus uses the V*δ*1 chain paired with different V*γ* chains, and these preferentially home in epithelial tissues as the intestine [9]. V*δ*1 T cells constitute only a minor proportion of human blood while they are a large population of the human intraepithelial cells and have been found to enrich various human epithelial tumors and lymphomas [10]. V*δ*1 T cells recognize stressed cells via presentation of self-lipids by CD1 and/or expression of stress-induced molecules through the NKG2D receptor. In contrast, V*γ*9V*δ*2 T cells are the major subset of the adult peripheral blood of humans, ranging from 80/90% of *γδ* T cell pool. They typically recognize phosphomonoester molecules synthesized in the mevalonate (MVA) and 1-deoxy-D-xylulose 5-phosphate (DOXP) metabolic pathway [11]. These lymphocytes have been defined “nonconventional” T cells, owing to several distinguishing features that are shared with both innate and adaptive immune cells. Since their discovery, *γδ* T cells have been shown to play a significant role against pathogens and tumors and they were placed in the innate immunity as cells of immunosurveillance. In antitumor response, *γδ* T cells show a high production of IFN-*γ* in the early development of tumor [12, 13]. *γδ* T cells may recognize and kill tumor cells through the engagement of NKG2D expressed on their surface which binds MICA and MICB or retinoic acid early-1, respectively, in humans and in mice. These ligands are up-regulated on several tumor cells including melanoma cells acting as target for their destruction by locally resident intraepithelial lymphocytes (IEL) as well as other cytotoxic lymphocytes. The binding can provide a costimulatory signal for cell lysis [14–16].

Several studies have reported *in vitro *reactivity of both human V*δ*2 and V*δ*1 against a broad range of tumor cell lines, normal cells infected with viruses, parasites, and bacteria [7]. In respect to transformed cells, the range of tumor cell lines recognized by V*γ*9V*δ*2 T cells is now extended to either haematopoietic or solid tumors [16–18]. Both subsets of human *γδ* T cells are able to recognize and destroy tumor cells as well as produce proinflammatory and Th1 cytokines through different mechanisms. A direct implication of these cells *in vivo *diseases is well accepted. However, distinct mechanisms in the recognizing and the functions of V*δ*2 and V*δ*1 T cells have been demonstrated, according also to different tumors, infections, and environments.

The highly restricted T cell receptor V region repertoire of *γδ* T cells is certainly one of the most salient features distinguishing these lymphocytes from conventional MHC restricted *αβ* T cells. Most V*γ*9V*δ*2 T cells react against the same related set of nonpeptidic, phosphorylated antigens [15, 16] that are produced by both microbial and endogenous metabolites, whereas V*γ*9V*δ1* T cells seem to recognize heterogeneous yet undefined antigens, presumably unrelated to V*γ*9V*δ*2 agonists. V*γ*9V*δ*2 T cell antigens are recognized in a TCR-dependent manner and are referred to as phosphoantigens. The most potent antigen appears to be the hydroxydimetihyl-allyl-pirophosphate (HDMAPP), an intermediate of DOXP pathway, restricted to plant cells and some microorganisms. Metabolic intermediates as isopentenyl pyrophosphate (IPP) can also activate V*γ*9V*δ*2 T cells although at concentrations of 100000-fold higher than those for microbial agonists. These compounds derive from the MVA pathway used by mammalian cells and some bacteria and are essential for sterol synthesis, cell growth, and membrane integrity. Aminobisphosphonate (ABPs) compounds may also stimulate V*γ*9V*δ*2 T cells through their ability to inhibit farnesyl pyrophosphate synthase, an enzyme acting downstream of IPP synthesis along MVA pathway, promoting intracellular accumulation of IPP. Finally, the alkylamines remain a debated class of antigens. Some studies strongly suggest that like ABPs, alkylamines promote intracellular accumulation of V*γ*9V*δ*2 agonists derived from MVA pathway [19]. Although phosphoantigen-mediated activation of V*γ*9V*δ*2 T cells clearly requires the expression of TCR, as indicated by gene transfer approach, how precisely this occurs remains unclear to date. The cell-cell contact required for the activation implicates either that phosphoantigens induce the structural modification of TCR or that are presented by surface molecules at present undefined. Interestingly, V*γ*9V*δ*2 T cells have been recently shown to recognize a complex formed between apolipoprotein A1 and ATP synthase, a mitochondrial enzyme that is translocated on the surface of normal hepatocytes and some tumor cell lines, in a TCR-dependent fashion [20]. Among other costimulatory factors, human V*γ*9V*δ*2 T cells express frequently activating or inhibitor NK receptors such as NKG2D or CD94/NKG2A that seem to be the major contributing receptor to V*γ*9V*δ*2 T cell activation/inhibition [21]. In the plethora of activation signals of human *γδ* T cells, an important pathway is represented by Toll-like receptors (TLR). TLRs have emerged as central regulators of innate immunity being receptors specifically sensing molecular patterns of microbes, leading to immediate cellular responses through the activation of transcription factors, notably NF-*κ*b, AP-1, and IRF [22]. Although certain TLRs are expressed on myeloid cells, several reports have shown functional expression on B, *αβ* and *γδ* T cells [23]. It has been reported that TLR ligands including TLR3 ligand poly (I:C) and TLR9 ligand CpG enhance the activation of *γδ* T cells *in vitro* via promoting type I IFN production in myeloid and plasmacytoid dendritic cells (DCs), respectively [22]. More recently, it has been shown that highly purified *γδ* T cells expressed more TLR3 mRNA than *αβ* T cells, thus opening the possibility that *γδ* T cells might respond directly to TLR3 ligands in the absence of APC [24]. Taken together, these results confirm that *γδ* T cells may play a crucial role in innate immune response and studies on the different signals activating or enhancing their functions may help to improve the understanding of these cells and their usage in the immunotherapy.

Although immunological memory is a hallmark of adaptive immune response, V*γ*9V*δ*2 T cells seem to show some features of memory cells. Studies in monkeys suggested that phosphoantigen-specific V*γ*9V*δ*2 T cells, expanded during a primary TB vaccination, showed an accelerated response after a secondary challenge [25]. The ubiquitous nature of exogenous and endogenous phosphoantigens for V*γ*9V*δ*2 T cells also suggests that the development of memory state may be quite different from conventional cells, which are programmed to respond to foreign peptide antigens. After antigen exposure, V*γ*9V*δ*2 T cells undergo the same change of CD8 T cells. Basing on the expression of CD45RA and CD27 molecules on their surface, it is possible to distinguish 4 subsets of V*γ*9V*δ*2 T cells as naïve, central memory (T_CM_), effector memory (T_EM_), and terminal differentiated effector cells (T_EMRA_). V*γ*9V*δ*2 T cells acquire CD45RO expression like early memory CD8 T cells and are termed central memory V*γ*9V*δ*2 T cells. They lose CD27 and CD28 expression and re-express CD45RA becoming terminally differentiated cells. Approximately 90% of V*γ*9V*δ*2 T cells in the adult have a memory phenotype [26]. Effector memory V*γ*9V*δ*2 T cells represent a readily available pool of antigen-primed V*γ*9V*δ*2 T cells which enter the peripheral tissues, where they can eventually further differentiate into CD45RA+CD27− cells, produce cytokines, and exert cytotoxicity contributing to the containment of invading microbial pathogens. 

In TB, the establishment of the disease and its clinical manifestations are closely linked to host's immune response. The spectrum of the immune response in TB ranges from a protective response in latent TB, to the absence of response and the dissemination of mycobacteria in miliary TB. In pulmonary TB, there is an effective antimycobacterial response with a clinically progressive disease involving innate and adaptive immune compartment [27]. MTB cannot evade the induction of cell-mediated immunity; MTB has evolved to survive it, and survive it does—even if the initial infection is successfully controlled, many infected individuals develop a latent infection that can persist for decades. On the other hand, some heavily MTB exposed individuals show no signs of infection: no pathology, no symptoms, and no apparent adaptive immune response. It is possible that in these cases, the innate immune response has eliminated the pathogen at the earliest stage [28]. Indeed, early immune mediators as IFN-*γ* are produced initially by NK cells and *γδ* T cells before adaptive T cells are instructed by IL-12 and IL-18 secreted by antigen presenting cells (APC) as DCs and macrophages. For this reason, we will focus the attention on *γδ* T cells that represent an optimal bridge between innate and adaptive immune response [29, 30]. Interestingly, the activity of a subset of human *γδ* T cells *in vitro* and *in vivo* can be stimulated by many nonpeptidic molecules (some drugs are currently being tested in Phase I cancer trials). The relatively low *in vivo* toxicity of many of these drugs makes possible novel vaccine and immune-based strategies for infectious diseases. Collectively this scenario indicates that different pathways and cell types interact to mediate innate immunity against MTB providing mechanisms that could likely be target for future therapeutic interventions in TB.

## 3. *γδ* T Cell Response in MTB Infection

In infections, responses of *γδ* T cells to MTB were described as early as in 1989 [31]. Later, a range of studies described a marked expansion of this subset in the blood of (TB) patients and also with a range of other infections as leprosy, malaria, salmonella, and *Streptococcus pneumoniae*. Mycobacterial phosphoantigens were identified as potent stimulators of V*γ*9V*δ*2 T cell functions [15]. Specifically, V*γ*9V*δ*2 T cells predominate in mycobacterial infections whereas V*δ*1 T cells are preferentially expanded in HIV patients and in immunocompromised subjects probably undergoing CMV reactivation [32, 33]. Parallel to the studies in murine models, an association between mycobacterial and human V*γ*9V*δ*2 T-cell responses was rapidly established. *γδ* T-cell clones were isolated from synovial fluid of rheumatoid arthritis patients, which had been stimulated with mycobacterial antigens and were found to proliferate to mycobacterial antigens [34]. *In vivo*, *γδ* T cells were observed in granulomatous skin reactions of leprosy patients, and *γδ* cell lines derived from these persons proliferated to mycobacterial extracts [35]. Direct evidence for the ability of MTB to activate *γδ* T cells was provided by studies of Kabelitz et al. which determined by limiting dilution analysis that the majority of peripheral blood *γδ* T cells proliferated in response to a killed preparation of MTB bacilli [36]. Subsequently, the predominance of V*γ*9V*δ*2 T cells in TB infection was confirmed [37]. Being MTB an intracellular pathogen residing within mononuclear phagocytes, many studies on the role of these cells in the activation of *γδ* T cells following the infection of MTB rapidly appeared. Monocytes infected with MTB were found to be efficient accessory cells for *γδ* T cells in a non-MHC restricted manner [38].* In vitro*, Havlir et al. demonstrated that monocytes infected with live MTB bacilli were particularly effective in expanding V*γ*9V*δ*2 T cells, compared to heat-killed bacteria and soluble protein antigens of MTB [39]. Similarly, MTB-infected alveolar macrophages, the first target of inhaled MTB, served as non- MHC-restricted accessory cells for *γδ* T cells. There were differences, however, between alveolar macrophages and monocytes. At the high alveolar macrophage to T-cell ratios normally present in alveolar spaces, expansion of resting *γδ* T cells was inhibited by alveolar macrophages in a dose- and cell-contact-dependent manner. However, upon invasion by MTB, alveolar macrophages are certainly capable of serving as accessory cells for *γδ* T cells, providing a mechanism for *γδ* T-cell activation in the lung. *γδ* T cells are dependent on costimulators for proliferation, cytokine secretion, and expression of cytolytic effector function. Like their *αβ*TCR+ counterparts, *γδ* T cells are dependent upon interactions of CD40-CD40L, CD28-B7.1/7.2, and CD2, as well as adhesion molecules (CD2-LFA-3, LFA-ICAM) for co-stimulation, and these second signals are readily provided by accessory cells such as monocytes and alveolar macrophages [40]. Whether accessory cells process and present mycobacterial antigens to MTB activated *γδ* T cells, and thus serve as true antigen-presenting cells, has yet to be determined. 

The major effector functions of T cells in the immune response to MTB are cytokine secretion, cytotoxic effector function (CTL), and cell-contact-dependent “help”. The goal of these effector functions is to help to contain mycobacterial growth and to stimulate memory immunity. Studies with MTB antigen-activated *γδ* T-cell clones or primary cells determined that there was some heterogeneity in cytokine profile among clones [41]. There was no clear-cut Th-1 versus Th-2 dichotomy, nor there were major differences found in cytokine patterns between *〈* *αβ* TCR+ and *γδ* T-cell clones*〉*. In general, most *γδ* T-cell clones produced IFN-*γ*, a cytokine associated with protective immunity to MTB, and a marker of the proinflammatory cytokine environment characteristic of the cellular immune response to intracellular bacteria. Some studies have used intracellular staining for cytokines and determined that in response to phosphoantigens, *γδ* T cells produce both TNF-*α* and IFN-*γ* [42]. When MTB-activated CD4+ and *γδ* T-cell populations from healthy tuberculin-positive donors were analyzed for patterns of cytokine production in response to MTB-infected monocytes, both subsets secreted large amounts of IFN-*γ* [43]. Intracellular IFN-*γ* levels were similar between CD4+ and *γδ* T cells, suggesting more efficient IFN-*γ* release by *γδ* T cells. In contrast, CD4+ T cells produced more IL-2 than *γδ* T cells, which correlated with diminished T-cell proliferation of *γδ* T cells compared with CD4+ T cells. CD4+ and *γδ* T cells from some healthy donors produced IL-4, reemphasizing the absence of a Th-1 versus Th-2 dichotomy among these two T-cell subsets. 

Although activated *γδ* T cells produce IL-2, they produce far less IL-2 than CD4+ T cells, which accounts for their poor proliferative ability and need for exogenous IL-2 to induce *γδ* T-cell expansion [44]. Furthermore, IL-15 is a T-cell growth factor for *γδ* T cells, and is produced by mononuclear phagocytes, thus providing a link between MTB-infected macrophages and *γδ* T-cell activation in the absence of CD4+ T-cell responses [42]. The balance between these two factors (CD4 versus macrophage “help”) may account for the variability one observes in *γδ* physiology. Finally, contribution of V*γ*9V*δ*2 T lymphocytes to immune protection against MTB is based also on the cytotoxic effector functions of these cells. It was reported earlier that V*γ*9V*δ*2 T lymphocytes kill macrophages harboring live MTB through a granule-dependent mechanism, resulting in killing of intracellular bacilli; moreover, it has been reported that these cells reduce the viability of both extracellular and intracellular MTB through granulysin and perforin, both detected in V*γ*9V*δ*2 T lymphocytes. These findings have suggested that V*γ*9V*δ*2 T lymphocytes directly contribute to a protective host response against MTB infection [45]. 

A controversial feature of V*γ*9V*δ*2 T lymphocytes is based on their capability to mount a memory response against a microbial reinfection or reactivation. V*γ*9V*δ*2 T lymphocytes mount a response against MTB infection during the early phases of infection, and a strong expansion of this T cell subset has been observed in different reports, but their functional response against mycobacterial infection seems not limited to an innate reaction. As mentioned above, V*γ*9V*δ*2 T cells follow a phenotype differentiation similar to *αβ* T cells and probably a certain memory response is generated. Relevant studies in mice cannot be performed because murine *γδ* T cells do not express the homolog of V*γ*9V*δ*2 TCR, and there is no functional equivalent for these cells, so far, identified in mice. A pioneering study showed the capability to mount a memory response after microbial reinfection or reactivation [25]. Indeed, to examine a primary role of *γδ* T cells during mycobacterial infection, macaques inoculated with BCG were analysed for the change in the *γδ* T cell repertoire. Striking expansion of V*γ*9V*δ*2 T cells were detected in the blood after BCG inoculation, whereas there was no apparent increase in other *γδ* T cell subsets. This expansion indicated the development of primary response of these cells during mycobacterial infection. Apart systemic V*γ*9V*δ*2 T cells, other pools of expanding V*γ*9V*δ*2 T cells have been observed in pulmonary and intestinal tissues after intravenous BCG inoculation and only a small amount of these cells were observed in lymph nodes suggesting that tissues but not peripheral lymph node tissues could expand in response to mycobacterial infection. Of note, after a second inoculation of BCG, a marked re-expansion of V*γ*9V*δ*2 T cells appeared in the blood. The expansion of these cells after a reinfection was 2–9 times larger than those seen during the primary infection. Furthermore, the expansion was persistent for as long as 7 months after the second BCG challenge [25]. This evidence provide that V*γ*9V*δ*2 T cells underwent polyclonal expansion during a primary mycobacterial infection can mount a memory recall response after a secondary challenge. Another important result has been reported by other reports suggesting that the route and the dose of mycobacterial infection related to the expansion of *γδ* T cells. Systemic BCG inoculation induced a dose-dependent expansion of circulating *γδ* T cells as well as CD4 and CD8 T cells whereas, in the pulmonary compartment, the systemic infection resulted in a predominant increase in numbers of *γδ* T cells. In contrast, pulmonary exposure to mycobacteria induced a detectable expansion of CD4, CD8, and *γδ* T cells only in the lung but not in the blood [46, 47]. The pattern and kinetics of *γδ* T cell responses during mycobacterial infection might contribute to characterizing immune protection against TB and testing new TB vaccines in primates.

## 4. *γδ* T Cells Cross-Talk with DCs during Mycobacterial Infections

Recent knowledge about the interaction between apparently different compartments of immune system changed the way to consider the immune response and its regulation. Studies in animal models suggest that even the smallest population of immune cells in a site of infection can exert large biological effects up to systemic level. This recent concept is due to the continuous interaction between local and recruited innate immune cells with APCs. Indeed, *γδ* T cells and DCs participate in early phases of immune response against MTB. Continuous cross-talk between *γδ* T cells and myeloid cells is evident in histological studies, *in vitro* culture experiments, and in animal models. Indeed, *γδ* T cells participate in early immune response against MTB producing cytokines (IFN-*γ* and TNF-*α*) and chemokines, prompting cytotoxicity or modulating other cell in mice [48]. 

The first evidence of an influence exerted by *γδ* T cells on DCs system came from studies by Ismaili et al., showing that human *γδ* T cells activated *in vitro* by phosphoantigens are capable of inducing maturation of monocyte-derived DCs [49] and this process involved both membrane-bound (i.e., CD40L) and soluble (i.e., TNF-*α* and IFN-*γ*) T cell-derived signals [49–51]. Recent studies support the notion that DCs strengthen the cellular immune response against mycobacterial infection [52–56]. Even if the critical role of DCs in the initiation of immune response has been firmly established [57], their involvement in immune responses occurring at sites of MTB infection needs further elucidations. DCs are highly represented at sites of MTB infection at the onset of the inflammatory response [58], and it is conceivable that immature DCs present in the lung mucosa are specialized for antigen uptake and processing. After interaction with pathogens, they mature and migrate to lymphoid organs where they prime naïve T cells through cell surface expression of MHC and costimulatory molecules and the secretion of immunoregulatory cytokines such as IL-12. 

Although infection with mycobacteria has been reported to induce maturation of DCs, *in vitro* infection of human mDC by virulent MTB strain H37Rv has been shown to impair their maturation, reduce their secretion of interleukin (IL)-12, and inhibit their ability to stimulate T cell proliferation [51, 59]. *In vivo *experiments demonstrated that MTB affects DC migration and antigen presentation, promoting persistent infection in mice [60]. These findings suggest that MTB can interfere with the host immune response by hampering several functions of DCs, and in particular suppressing the migration of mDCs and modulating its cell trafficking ability, and these mechanisms may encourage the long-term persistence of the bacilli in the host during chronic infections. However, in more physiological situation, such as during infection by a V*γ*9V*δ*2-stimulating pathogen unable to promote complete DC maturation, it is easy to hypothesize that many different stimuli, besides microbial-derived phosphoantigens, may influence the activation state of DCs and V*γ*9V*δ*2 T cells. Accordingly, in a recent paper, Meraviglia et al. demonstrated that V*γ*9V*δ*2 induce full maturation of MTB-infected immature DCs, that were otherwise unable to complete maturation. In detail, MTB infection caused up-regulation of CD86 and HLA-DR molecules, but not of CD80 and CD40, while the co-culture of MTB-infected DCs with V*γ*9V*δ*2 T cells determined up-regulation of CD80 and CD40 expression, no changes of HLA-DR and CD86 expression, and a significant up-regulation of IL-12p70 production, suggesting that V*γ*9V*δ*2 T cells mediate full maturation of MTB-infected DC [61]. On the other hand, MTB infected DCs lead to a rapid and strong activation of co-cultured V*γ*9V*δ*2 T cells without requirement for any additional stimulations. The MTB infected DC-mediated potentiation of V*γ*9V*δ*2 T cell responses could be explained at least in part by up-regulation and/or presentation of MTB derived phosphoantigens to V*γ*9V*δ*2 T cells. However, and most surprisingly, MTB infected DCs selectively induced proliferative, but not cytokine or cytolytic responses by V*γ*9V*δ*2 T cells and this was associated to the expansion of phenotypically “immature”, central memory-type V*γ*9V*δ*2 T cells. Similar results have been obtained using BCG infected DCs co-cultured with V*γ*9V*δ*2 T cells [62]. Possible explanation for the incomplete phenotypic and functional differentiation of V*γ*9V*δ*2 T cells include the lack of IL-15 production by MTB infected DCs; IL-15 is a relevant cytokine for the differentiation of *γδ* cells [63], and its main effect in the pathway leading to differentiation of V*γ*9V*δ*2 T cells towards effector memory cells was associated with induction of Bcl-2 expression and resistance to cell death [63]. Indeed, adding IL-15 to co-cultures of MTB infected DCs and V*γ*9V*δ*2 T cells caused efficient differentiation of *γδ* T cells with maintenance of the central memory pool and with generation of effector-memory and terminally differentiated effector memory cells, which displayed potent antimycobacterial function, as demonstrated by their ability to efficiently reduce the viability of intracellular MTB. We therefore conclude that mechanisms fine-tuning the DC-*γδ* T cells cross talk are still not clear, including identification of the critical receptor/ligand interactions, as well as the underlying molecular mechanisms; therefore, further studies are needed such as the analysis of the effect of inhibitors of various signalling cascades coupled with transcriptome analysis of maturing DCs at various time points after V*γ*9V*δ*2 T cell incubation.

## 5. Incomplete Maturation of *γδ* T Cells in TB Patients

A number of studies have attempted to determine the *in vivo* role of *γδ* T cells in the human immune response to MTB. Barnes et al. established that patients with pulmonary or miliary TB had a diminished ability to expand *γδ* T cells *in vitro *in response to heat-killed MTB and IL-2, although there was quite a range of *γδ* T-cell expansion among the different groups [64]. Some investigators have suggested an increase in peripheral *γδ* T cells in patients with TB or among hospital workers with contact with TB patients but this has not been a consistent finding [65, 66]. Studies of T-cell phenotype in bronchoalveolar cells from healthy PPD^+^ subjects and from affected and unaffected lungs of patients with pulmonary TB found a T lymphocytic alveolitis in the affected tuberculous lung [67]. *γδ* T cells were found among the lymphocytes in this alveolitis, but their proportion was not increased relative to *αβ* T cells. Thus *γδ* T cells are present *in situ* in pulmonary TB but are not expanded compared to *γδ* T cells in peripheral blood or unaffected lung. The presence of *γδ* T cells in the tuberculous lung is consistent with the findings that alveolar macrophages can serve as APCs for *γδ* T cells. In the study by Schwander et al., monocytes were markedly increased in alveolar spaces of tuberculous lung [67]. Monocytes also are efficient APCs of *γδ* T cells, and hence in TB adequate accessory cell populations are available for *γδ* T-cell activation.

More recently, dramatic expansion of V*γ*9V*δ*2 T cells has been found after BCG vaccination in infants, and several phosphorylated antigens derived from mycobacteria have been defined [68]. It is already known in the context of a natural infection the consistent expansion of V*γ*9V*δ*2 T cells with a T_CM_ phenotype in the peripheral blood of patients with active TB, which was accompanied by the dramatic reduction of the pool of V*γ*9V*δ*2 cells with immediate effector functions (T_EM_ and T_EMRA_ cells). However, this skewed representation of circulating V*γ*9V*δ*2 T cell phenotypes during active TB was transient and completely reversed after successful antimycobacterial therapy. This explains previous findings from Dieli's group, showing that V*γ*9V*δ*2 T cells from children affected by active TB have an increased proliferative activity, but decreased IFN-*γ* production and granulysin expression [69]. After successful chemotherapy, the V*γ*9V*δ*2 T cell proliferative response strongly decreased, whereas IFN-*γ* and granulysin production consistently increased. 

Other previous observations have indicated an increased proliferative activity of V*γ*9V*δ*2 T cells from patients with TB [70, 71] but reduced production of IFN-*γ*, compared with that of healthy tuberculin reactors [72]. Additionally, Dieli et al. reported that decrease of V*γ*9V*δ*2 T cell effector functions involves not only IFN-*γ* production but also expression of granulysin, a molecule known to be responsible for the killing of MTB [69]. The reason for the loss of V*γ*9V*δ*2 T cell effector functions during TB is unknown. One possibility is that sustained *in vivo* mycobacterial stimulation of V*γ*9V*δ*2 T cells causes their apoptosis [73]. For example, high levels of bacteria (such as those occur in patients with TB), resulting from the inability to contain and prevent their spread, would presumably result in chronic stimulation of effector V*γ*9V*δ*2 T cells by mycobacterial antigens and in their apoptosis, thus providing an explanation for why this population of *γδ* T cells is lost in patients with active disease but recovers after drug therapy. Alternatively, it is possible that reduced IFN-*γ* and granulysin expression in children with TB, which recovers after disease improvement, could be the consequence of generalized illness. The finding that IFN-*γ* and granulysin production are restored by successful chemotherapy, which is suggested to induce the generation of a protective immune response, strongly supports this possibility. Another possible explanation for the incomplete phenotypic and functional differentiation of V*γ*9V*δ*2 T cells could be explained by the lack of relevant cytokines secreted by MTB infected DCs. In fact, it has been previously shown that, differentiation of V*γ*9V*δ*2 T_CM_ cells into T_EM_ and T_EMRA_ cells occurs upon antigen stimulation in the presence of IL-15, while any other tested cytokine, including IL-7, had no such effect [63]. The main effect of IL-15 in the pathway leading to differentiation of V*γ*9V*δ*2 T cells towards effector memory cells was associated with resistance to cell death and Bcl-2 expression. Meraviglia et al. demonstrated that the lack of IL-15 production by MTB infected DCs was not due to the fact that MTB simply does not induce synthesis of this cytokine, rather it actively inhibits IL-15 secretion. Additionally, and similar to the *in vitro* data, their analyses of IL-15 serum levels in healthy contact (HC) subjects and TB patients showed that IL-15 production is not induced in patients with active TB, but increases after completion of chemotherapy [61]. However, the analysis of V*γ*9V*δ*2 T cell functions in TB patients and especially in the site of infection needs further investigations.

## 6. *γδ* T Cells Producing IL-17: Possible Involvement in Mycobacterial Infection

The cytokine IL-17 has received considerable attention since the discovery of a distinct CD4+ T helper cell subset producing it, known as Th-17 profile. This discovery provided compelling reasons to explore outside the Th-1/Th-2 paradigm, searching new answers to explain independent effector T cell responses. A rapid succession of studies defined the Th-17 cell paradigm, in which IL-6/STAT3 activation of the transcriptional regulator retinoic acid receptor-related orphan receptor-*γ*t (ROR*γ*t) controls the lineage fate of IL-17A-, IL-17F-, IL-21-, and IL-22-producing T cells (collectively known as Th-17 cells) that are highly responsive to IL-1 receptor 1 (IL-1R1) and IL-23R signaling [74]. IL-17A has been reported to participate in host defense against various types of pathogen [75, 76] and estimated to be an important cytokine in the immune response against mycobacterial infection [77]. Indeed, IL-17 is produced immediately after pulmonary BCG infection and was also detected at later stages of MTB infection in mice [78]. Interestingly, IL-17-expressing cells in the mycobacterial infected lungs in murine models are *γδ* T cells rather than CD4+ T cells. As mentioned, *γδ* T cells may play an important role in the effector functions and regulation of immune responses to infection of MTB, but the precise role of IL-17 producing *γδ* T cells remains unclear. In a study performed on 27 patients with active pulmonary TB and 16 healthy donors, it has been found that proportion of IL-17-producing cells among lymphocytes was similar between TB patients and HD, whereas the proportions of *γδ* T cells in IL-17-producing cells (59.2%) in peripheral blood were markedly increased in TB patients when compared to those in HD. In addition, the proportions of IFN-*γ* producing *γδ* T cells in TB patients were obviously lower than that in HD. Upon re-stimulated with MTB heat-treated antigen *in vitro*, fewer IL-17-producing *γδ* T cells were generated from HD than TB patients [79]. These findings were consistent with murine investigations showing that the IL-17-producing *γδ* T cells were main source of IL-17 in mouse model of BCG infection, suggesting that *γδ* T cells might be involved in the formation of tubercular granuloma in pulmonary TB patients [80], but these investigations need further identification in humans. Another study showed the differentiation and distribution of human IL-22-producing T cells in a nonhuman primate model of MTB infection. Since IL-22-producing T cells also produce IL-17, knowledge about this cytokine profile may help the understanding of the Th-17 profile. An apparent increase in the number of T cells capable of producing IL-22 de novo without *in vitro* Ag stimulation has been observed in lungs compared to blood and lymphoid tissues. Consistently, IL-22-producing T cells were visualized *in situ* in lung TB granulomas, indicating that mature IL-22-producing T cells were present in TB granuloma. Surprisingly, phosphoantigen HMBPP activation of V*γ*9V*δ*2 T cells down-regulated the capability of T cells to produce IL-22 *de novo *in lymphocytes from blood, lung/BAL fluid, spleen and lymph node. Up-regulation of IFN-*γ*-producing V*γ*9V*δ*2 T effector cells after phosphoantigen stimulation coincided with the down-regulated capacity of these T cells to produce IL-22 *de novo* [81]. These findings raise the possibility to ultimately investigate the function of IL-22 producing T cells and to target V*γ*9V*δ*2 T cells for balancing potentially hyper-activating IL-22-producing T cells in severe TB.

## 7. *γδ* T Cell-Specific Phosphoantigen Based-Immunotherapy in TB: Lesson from the Cancer

As mentioned above, *γδ* T cells have antimicrobial as well as antitumor activity through the production of proinflammatory cytokines, chemokines, and cytotoxic molecules such as perforins and granzymes. This suggests their involvement in the control of infections *in vivo* and could be considered as target for new intriguing therapeutic approaches. Moreover, the capacity of *γδ* T cells to interfere in DC functions would allow their use in specific immunotherapy. Although other nonclassical lymphocytes may support DC maturation and contribute to the antigen presentation, *γδ* T cells in humans represent an easy model to amplify the DC system. Given that the different classes of pharmacological agents are used in therapies for different diseases, the possibility to make new vaccines or adjuvants based on these compounds is very close. A variety of natural and synthetic nonpeptidic antigens have been demonstrated to activate *γδ* T cells such as IPP, dimethylallyl diphosphate (DMAPP), geranylgeranyl pyrophosphate (GGPP) including Nitrogen containing bisphosphonates (N-Bps). At present two approaches showed exciting results. Tumor immune evasion mechanisms are common and include the down-regulation of tumor-associated antigens, MHC, and costimulatory molecules. By contrast to *αβ* T cells, *γδ* T cells are not MHC restricted and show less dependence on costimulatory molecules such as CD28. Moreover, *γδ* T cells are involved in the resistance of cutaneous carcinogenesis in mice and display potent cytotoxicity against various human tumor cell lines *in vitro*. Indeed, human V*γ*9V*δ*2 T cells expanded *in vitro *and transferred to immunodeficient mice, xenografted with tumor cells, showed efficacy against B cell lymphoma, melanoma, and renal carcinoma [82]. On this ground, in patients with multiple myeloma or with low-grade non-Hodgkin lymphoma, occurrences of acute phase reaction to intravenously injection of an aminobisphosphonate, called Pamidronate (PAM), were attributed to the systemic activation of *γδ* T cells [83], and this provoked the deliberate treatment of lymphoma patients with PAM and IL-2. Promising results were achieved after the patients were prescreened for substantively response to PAM and IL-2 of *γδ* T cells *in vitro*. By several criteria, zoledronate is more potent and efficacious than PAM. Previous studies in patients with breast and prostate tumors showed that zoledronate induced *in vivo *activation of peripheral *γδ* T cells into more potent cytotoxic and IFN-*γ* producing cells. Recently, a phase I clinical trial in metastatic HRPC has been conducted by Dieli et al. to determine the safety, feasibility, and response induced by V*γ*9V*δ*2 T cells *in vivo*, using zoledronate alone or in combination with low-doses of IL-2 [84]. The encouraging prospect that the activation of peripheral blood V*γ*9V*δ*2 T cells can be efficacious against solid tumors could be explained by the double role played by these cells; activated *γδ* T cells can infiltrate tumor sites and display cytotoxic activity against tumor cells or they help other cells as DCs to trigger an adequate specific CD8 T cell immune response.

Different interesting results have been shown in animal models aimed to improve the effectiveness of vaccination against TB. As known, although the vaccination with BCG protects children against disseminated TB, it is now clear that it does not protect efficiently against pulmonary disease. Therefore, the ever-increasing incidence of TB worldwide urges to improve this vaccine. It is widely accepted that one of the best immunological predictors of protective and long-lasting immunity to TB is a high frequency of MTB-specific IFN-*γ*-secreting cells (ISCs) in the peripheral blood [85]. A quantitatively sizeable population of effector T cells able to release IFN-*γ* seems to promote the protective bioactivity of infected macrophages. Therefore, most of current TB vaccine candidates and injection regimens aim to increase the frequency of these MTB-specific ISC. These candidates comprise recombinant BCG, attenuated MTB, modified vaccinia virus, naked DNA, and subunit combinations of either MTB protein antigens or recombinant fusion proteins [86]. It exists now a consensus on the ability of heterologous prime-boosts regimens to induce high titers of MTB-specific ISC. The priming with an optimized “starter” such as BCG or improved BCG could likely induce a broad diversity of memory cells. The further boost with antigens common to the priming would expand and differentiate into effector memory, MTB-specific ISC. The immunodominant protein antigens from MTB include members of the “proline-proline-glutamic acid family” proteins (Mtb39a-e), Mtb9.9, TB10.4, so-called “6-kDa early secretory antigenic target” (ESAT-6), and mycolyl transferase complex Ag85A, B, C [86]. Despite their good specificity, these purified antigens were weakly immunogenic when injected alone, and therefore needed to be combined either to other antigens (hybrid proteins) or to adjuvants. Hybrids of the most promising proteic antigens, namely, Mtb72F and H-1 have been generated: they corresponded to fusions Mtb39 to Mtb32 and ESAT-6 to mycolyl transferase complex antigen 85B (Ag85B), respectively. Hybrid H-1 is highly specific of MTB and induces a detectable ISC population but its immunogenicity was quite low, even after several boosts. Therefore, H-1 was combined with adjuvants such as Lipovac or IC31. Nonhuman primates *γδ* T cells like those from rhesus macaques present a TCR with similarity of 90% to the human V*γ*9V*δ*2 TCR sequence, and the same pattern of specificity for phosphoantigens [44]. Therefore, these animals represent a model suited to investigate the role of phosphoantigen-induced *γδ* T cell responses in immunity to TB. A pioneering analysis of rhesus infected with MTB demonstrated that rhesus *γδ* T lymphocytes mounted memory responses to mycobacteria. This adaptive response correlated with a faster *γδ* T cell expansion in the secondary respect to primary exposure to mycobacteria and was associated with a reduced bacteremia and protection against fatal TB. Furthermore, two studies have independently shown that blood *γδ* T cells from several monkey species could be monitored using mAb reagents for human T cells. These studies confirmed that phosphoantigen-induced proliferation of naïve, central memory CD27+, and effector memory CD27− *γδ* T cells require IL-2 *in viv*o [87]. Since macaque *γδ* T cells seem to react as human V*γ*9V*δ*2 T cells during BCG vaccination or TB infection and to phosphoantigen stimulation, the bioactivity of a synthetic phosphoantigen combined to a subunit vaccine candidate for TB has been assessed *in vivo*. Since TB mainly alters cytokine production and cytotoxic activity but not proliferation of human *γδ* T cells, this study focused on effector functions in defence against TB: secretion of Th-1 cytokines, most notably IFN-*γ*, and perforin [88]. In this paper, an efficient immunogenicity against MTB antigens in naïve cynomolgus after a prime-boost with the hybrid H-1 solubilized in Lipovac adjuvant with or without the synthetic phosphoantigen Picostim has been reported. Although the IC31 adjuvant was selected for clinical trial of the H-1 subunit vaccine, in this work the adjuvant Lipovac was preferred for its lower bioactivity, in order to be able to detect additional adjuvant effect on phosphoantigens. However, Picostim, a new generation of synthetic phosphoantigens, induced immediate cytokine production by *γδ* T cells (IL-2, IL-6, IFN-*γ*, and TNF-*α*), but a subsequent anergy up to 4 months after the initial administration. This phenomenon could be related to the TCR down-modulation/regulation or apoptosis induced cell death [73, 89]. However, this early *γδ* response translates into differential induction of recall response eliciting the H-1-specific *αβ* T cell responses, which essentially comprised recall of cytotoxic *αβ* T lymphocytes specific for Ag85B and few ISC *αβ* T lymphocytes in both groups of animals. So this study demonstrated that a prime-boost regimen with the H-1/phosphoantigen combination added a primary wave of adaptive immune responses from phosphoantigen-specific *γδ* T cells to the secondary wave of H-1-specific *αβ* T cells. In summary, nonhuman primates vaccinated with phosphoantigens associated to a subunit of antituberculosis vaccine, mounted a differential immune response by *αβ* or *γδ* T cells, where boosts anergized *γδ* T cells but promoted *αβ* recall responses. Finally, these models of usage of phosphoantigens against tumors and infections may allow to design subunit combinations promoting memory by both classes of lymphocytes in order to improve TB therapy.

## 8. Concluding Remarks

*γδ* T cells appear to combine properties of both adaptive and innate immunity. The identification of unusual compounds that are recognized by human *γδ* T cells but not by *αβ* T cells has recently stimulated great interest in the development of *γδ* T cell-based therapies. In contrast to other potential effector cells, it is possible to envisage combined *in vivo *activation and adoptive cell therapy with *ex vivo *expanded *γδ* T cells, because several drugs as ABPs and synthetic phosphoantigens are licensed for clinical application and in clinical trials, respectively. Recent advances on their multipotent functions, not only to the innate immune response, but to DC and antigen presentation system, increase the interest in a possible usage in clinical treatments. The effectiveness of these compounds in stimulating a cytotoxic response against tumors, as well as amplifing the antigen presentation of soluble specific peptides through DCs, represents a new possibility in the approaches based on immune cells. 

Furthermore, the intriguing capacity of *γδ* T cells to naturally respond to particular infections, such as TB, put these cells in a central place mainly in those pathologies where the classical presentation of antigens is compromised. However, *γδ* T cells are part of the multicellular immune system that is tightly regulated by multiple pathways and cells including the regulatory cells. We still know very little about the nature of *γδ* T cell antigens, their precise recognition mechanism, and their therapeutic relevance. Also the mechanisms regarding DC/*γδ* T cells cross talk are still not clear, as the receptors and ligands involved in this interaction, the molecular factors, and the possibility to verify this interaction in a model *in vivo*. Future studies should also address the possible advantage of combining *γδ* T cell therapy with conventional therapy or other therapeutical approaches.
